# Cyclists and autonomous vehicles at odds: Can the Transport Oppression Cycle be Broken in the Era of Artificial Intelligence?

**DOI:** 10.1007/s00146-022-01538-4

**Published:** 2022-07-19

**Authors:** Alexander Gaio, Federico Cugurullo

**Affiliations:** Department of Geography, Trinity College, Dublin, The University of Dublin, Dublin, Ireland

**Keywords:** Cycling, Artificial intelligence, Autonomous vehicles, Urban mobility, Vulnerable road users

## Abstract

Consequential historical decisions that shaped transportation systems and their influence on society have many valuable lessons. The decisions we learn from and choose to make going forward will play a key role in shaping the mobility landscape of the future. This is especially pertinent as artificial intelligence (AI) becomes more prevalent in the form of autonomous vehicles (AVs). Throughout urban history, there have been cyclical transport oppressions of previous-generation transportation methods to make way for novel transport methods. These cyclical oppressions can be identified in the baroque and modernist periods, and a *third oppression* may occur in the contemporary period. To explore the idea of a *third oppression*, we focus on the bicycle and outline the history of cycling to understand how historical mode oppression unfolded. We then present several social and political factors that contributed to the oppression of cycling and share recommendations for how to avoid future oppressions including political, social, and design actions for researchers and policymakers to take. This paper argues that priorities for AI-enabled mobility and cyclist needs be advanced in proportion to the extent that they contribute to societal goals of urban containment, public realm, and proximal cities. Additionally, future mobility evolutions should prioritise mobility justice and mode choice over inducing a singular transportation method.

## Introduction

Autonomous vehicles (AVs) driven by artificial intelligence (AI) were once a distant vision for mainstream adoption. Recent advances in AI technology and early testing have made the prospects of widespread adoption far more realistic (Acheampong et al. 2021; Milakis et al. 2017). The rise in ubiquity of AVs will usher in a new era for transportation systems. As we can learn from historical technological advances in transportation, it may bring about oppression of other transport modes, particularly cycling. This would lead to a reduction of choice in transport options. A reduction in transport options counteracts societal goals where cycling can address challenges related to climate change, public health, and traffic congestion (Garrard et al. 2012; Götschi et al. 2016; Kelly et al. 2014; Macmillan et al. 2014; Oja et al. 2011). Considering that cycling is a burgeoning and essential part of transport systems, its cyclical oppression is representative of historical trends caused by the coercion of novel transport technology (Prati et al. 2017; Urry 2004). This paper will apply a critical lens to the burgeoning realm of AI-enabled transport and the possible impacts on sustainable transportation behaviour, especially as it relates to individuals choosing to cycle for their transport needs.

Throughout urban history, there has been a cyclical oppression of previous-generation transportation methods to make way, often exclusively, for novel transport methods (Cugurullo et al. 2020). Broadly speaking, this cyclical oppression of previous-generation transport methods can be observed as cities have evolved. As identified by Cugurullo et al. (ibid), the cycles of oppression arose in three evolutionary approaches to city and state politics through the baroque, modernist, and contemporary periods. The often-overlooked political context in which each of the eras was predicated influenced how certain transport modes were prioritised and how others were oppressed (Cugurullo et al. 2020). For example, in the Baroque city, during the seventeenth century, pedestrians were oppressed to make way for the stagecoach as advanced by aristocratic politics. In the Modernist city, the car dominated public rights-of-way and prevailed over all other modes of transportation (Cugurullo et al. 2020; Hall 2002). Borenstein et al. (2020) highlight the divide between novel and previous-generation transportation methods through a debate between ‘occupant’ and ‘non-occupant’ safety where ‘occupants’ are the future users of AVs and ‘non-occupants’ are those that interact with AVs as other road users.

Bicycles have fallen victim to this cyclical oppression. Bicycle travel behaviour is influenced by a sense of comfort and safety that depends on the infrastructure extended to them, e.g. a grade-separated cycle track is preferential to a painted bike symbol on a busy street (Caulfield et al. 2012; Garrard et al. 2008). Compromising safety and comfort dissuades individuals from cycling. Later, we explore two real examples where bicycles were removed from transportation planning policy. Furthermore, by pitting vulnerable transport methods such as cycling (non-occupant) against AI-enabled (occupant) transportation, it hampers the desirable positive externalities that cycling yields on a population level. We fear and argue that similar oppressions might soon recur in the contemporary city as the diffusion of AVs increases, potentially to the detriment of other modes of urban transport. As history often repeats itself, this paper draws upon the history of urban transport to (a) develop a series of critical lessons about transport oppressions and (b) apply these lessons in the context of AI-mediated transport systems.

**Fig. 1:**
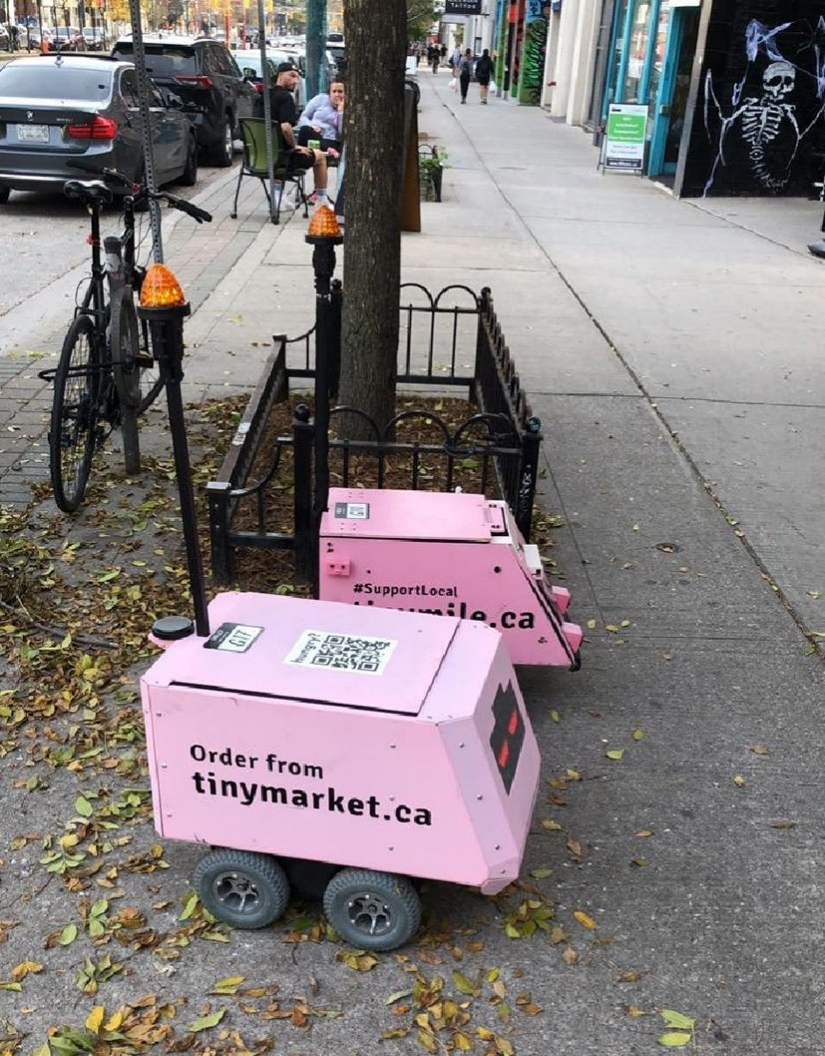
AI-enabled sidewalk delivery vehicles in Toronto. Source: Authors’ original

Cycling is not the only transport mode that stands to be impacted by AI-mediated transport systems. In many ways, other vulnerable road users (VRUs) such as pedestrians and micro-mobility users (skateboards, roller skates, and wheelchairs) will experience many of the same impacts that cycling will. However, they further stand to be impacted as AI-mediated transport systems encroach on sidewalks as demonstrated by the proliferation of adjacent applications such as dockless e-scooters and sidewalk delivery vehicles (Fig. 1). Public transport, also subject to historical subjugation, stands to be similarly impacted. In direct competition with transit, *shared* autonomous vehicles (SAVs) may be able to offer a greater value proposition (McCarroll and Cugurullo 2022). Instead of each autonomous vehicle being privately owned, SAVs are either collectively owned or deployed via sharing services such as ride-hailing companies, public transport operators, or transportation network companies (TNCs). If implemented, foresight analyses indicate that it would lead to a net decrease in private car ownership thereby reducing the number of cars in cities (ibid). This is corroborated by findings from many other scholars (Fagnant and Kockelman 2014; Firnkorn and Müller 2015; Haboucha et al. 2017; Iacobucci et al. 2018). A city with fewer cars leads to a safer city for VRUs and creates more space for people. Cugurullo et al. (2020) explain that this presents a unique window of opportunity to repurpose some 80% of space in cities that is currently reserved for cars. The proliferation of SAVs is likely to be from TNCs (Gurumurthy et al. 2020). Taken together, there are many different transportation modes that could be disruptors or be disrupted. (S)AVs present a generational opportunity to redefine cityscapes. Automobility has demonstrated its ability to shape cities for a single mode of transportation that is unsuitable for many urban trips and contributes to many negative externalities. Urban space and infrastructure priorities can be realigned for the provision of green space, additional housing, pedestrian plazas, active transportation, and urban agriculture, for example.

When funded by venture capital, in adjacent applications, there is little interest in infrastructure investment (Chambers 2020). This funding model also risks undercutting and driving competition away (Brown 2020; Dudley et al. 2017). The implications to public life and the privatization of the city advanced by the “capitalistic interests of competing private companies” (Cugurullo 2021, p. 56) need to be contemplated if regulators pave the way for this kind of new mobility. All said, these arguments form the basis of our critical lens and in this paper, we will focus on the impacts to cycling. However, the broader transportation system implications that AI-mediated transport will have cannot be understated.

The remainder of this paper is divided into five sections. First, we outline a brief history of cycling and its relationship with the evolving realm of AI-enabled mobility. Second, we define AI systems as it pertains to bicycle use. Third, we explore two case studies of bicycle oppression and resurgence. Fourth, we discuss some approaches that can be employed to avoid subsequent oppressions. Penultimately, we discuss how policymakers and researchers can play a role in avoiding future mode oppression. Lastly, we conclude with reflections for AI-enabled mobility and remark on broader societal implications.

## A brief history of cycling and mode subordination

Cyclists and pedestrians have only recently been afforded the space to reclaim a small portion of transportation demand as cities seek to advance policies to tackle the global climate crisis (Brand et al. 2021; Lanzendorf and Busch-Geertsema 2014; Maibach et al. 2009). The effects and political will to build out space for bicycles and simultaneously roll back oppressive policies (and related infrastructure) have created a window where bicycles are considered pragmatic and essential components of a complete urban transport network. This contrasts with historical and auto-oriented transportation planning principles that enabled systemic subordination of all transport besides the dominant mode, the automobile, when the Modernist city movement was initially conceived at the turn of the last century (Hall 2002).

Bicycles and automobiles were both invented in the nineteenth century. Motorists and cyclists initially were united in advancing a vision of paved road surfaces, however, the former ultimately excluded the latter from the very roads they collaborated on improving (Schwartz and Kelly 2018). This ‘partnership’ was not earnest and ended in a zero-sum outcome. Cars gained; bicycles lost. This should be a lesson learned as AVs mature so that another systematic oppression in the contemporary city can be avoided altogether.

Cyclical transport mode oppressions were the result of the political agendas, histories of infrastructure, societal norms, technologies, energy access, and geopolitical alliances. While this is not an exhaustive list, many forces influence transportation cycles. In our argument, we focus on the political context. The systematic segregation of slower moving traffic into designated spaces such as bus lanes, sidewalks, or bike lanes was taken to provide a higher standard of safety (Tefft 2013). The narrative of ‘safety’ that persists today was created by the early-twentieth century automobile industry that colluded with public officials to change the narrative from holding motorists accountable for inflicting danger on VRUs to holding them accountable for putting themselves in harm’s way. This led to the creation of the moniker “jaywalker” as a derogatory term for a VRU who ‘deliberately’ endangered themselves by walking into the street (Lewyn 2017). This is an example of victim-blaming (Pimentel 2017). The position advanced by the automobile industry was that it should be self-evident that streets were made for cars only to pass the burden of responsibility onto the VRU who would need to take precautions (Norton 2007).

Transport systems shifted their priority throughout history from walking and simple carts to horse-drawn carriages and stagecoaches. More recently bicycles, streetcars, and automobiles form part of the transport system. Often, when thinking about streets and roadways there is an inherent association with the automobile. Urry coined this concept as “the ‘system’ of automobility” (Urry 2004, p. 27) where it is explained that social life was “irreversibly locked into the mode of mobility that automobility generates and presupposes.” (ibid, p. 27). While presupposed, Urry further writes that “[t]his mode of mobility is neither socially necessary nor inevitable but has seemed impossible to break from” (ibid, p. 27). Several factors can be attributed to this, however, one of the biggest factors is the degree of *speed* and *flexibility* it enables, thereby coercing people into a system of intense flexibility (ibid) without regard for the negative externalities or the mode choice it severed.

Public transport was subject to a similar subordination. Streetcar suburbs of post-war suburbia became automobile suburbs when cars became cheap and prolific due to the low cost of oil. Automobility also afforded an individual nearly unfettered freedom. Furthermore, cities cemented private vehicle norms in rules such as minimum parking requirements, and bundling the cost of parking with housing. Shoup (2011) details the negative implications of parking oversupply and wider negative externalities automobility*.*

The next era in transport evolution is unfolding in the present one. The contemporary city is prompting countries like the US, UK, and the Netherlands to develop policies in support of AV deployment (Cugurullo et al. 2020). Of these countries that are advancing policies to support AV deployment, there are notably different infrastructure and political conditions that accommodate. This is made apparent by data reported by Buehler and Pucher (2021b) on the number of cyclist deaths per 100,000 population where the US reports far more deaths than the UK or the Netherlands.

## Defining AI systems from a bicycle-user perspective

AI systems will change the human-scale transportation landscape (Berge et al. 2022; Dey et al. 2020). Cutting-edge mobility enabled by AI is trained by human intelligence (Awad et al. 2018) and based on current traffic scenarios (see Best et al. 2018; Zhang et al. 2014). The simulations that are used to train AI are developed using the current state of automobility, which may not be the most suitable scenario to extrapolate to AI systems for VRU interaction, given the current power dynamic. Although AV vendors and AI-system suppliers stress their altruistic nature when interacting with bicycle users (see Waymo 2021), the existing infrastructure and transportation system baked-in to AI systems that will pilot AVs is problematic, as discussed later in this section. All things considered, by training AI systems to continue to behave in the current landscape of marginalised VRUs, AI will be acclimated to and may underpin contemporary bicycle oppression.

As it currently stands, AI-enabled mobility, specifically AVs, brings to light several challenges. AV capability is often described in a zero to five capability matrix as outlined by the Society of Automotive Engineers (2018) where level zero is no driving automation and level five is full driving automation. Levels three to five are sometimes referred to as ‘self-driving’. In some cases, self-driving is only used to describe vehicles that have capabilities in line with levels four and five (Tabone et al. 2021). The concern for another oppression lies in the capabilities of level three and higher if sensing capabilities and bicycle detection innovation remain complacent. In this scenario, segregating transport modes and engineering predictability into street design for immature autonomous detection technology could lend itself to a regressive *third oppression* akin to what happened in the Baroque city and the Modernist city. Segregating bicycles and other VRUs to accommodate early detection technology would further restrict the places and spaces where organic urban life can happen. Further to this, AV–cyclist interaction is not a common research topic (Hagenzieker et al. 2020) and this is pertinent given that bicycle detection is “probably the most difficult detection problem that AV systems face” (Fairley 2017: no page) and several challenges remain (Mannion 2019). This is because “they are relatively small, fast and heterogenous. […] A bicycle has much less mass and also there can be more variation in appearance” (ibid, no page). Furthermore, the datasets that AI use to train themselves have images that have concentrated on cars and the lack of bicycle images (ibid). This is corroborated by several studies that identify cyclist detection as a weak point (Ahmed et al. 2019a, b; Botello et al. 2019; Masalov et al. 2019; Pyrialakou et al. 2020; Zhang et al. 2020). AV safety systems need to earn the trust of society as well as understand all the ticks and quirks of erratic behaviour such as track stands, sudden manoeuvres (i.e. changing lanes), and precursors to actions i.e. riding out of the saddle to accelerate. The need for more research to demonstrate AV trustworthiness with cyclists is underdeveloped and should be more prominently emphasised.

There are emerging technologies that show potential to improve and/or benefit safety for VRUs. One such technology is the Human–machine interface (HMI) which acts as a beacon for AI-enabled mobility that relies on an Internet-of-things (IoT) architecture to make a living being part of a connected digital network where vehicles increasingly communicate through connected means (Berge et al. 2022). This is sometimes referred to as ‘vehicle to everything’ (V2X) communication (Dasanayaka et al. 2020). While research suggests that this technology can improve safety, a study by Winzer et al. (2019) shows that it would be rejected by potential users, therefore, weakening its overall effect on safety. It may also be useful to apply a critical lens when considering the *addition* of technology to solve a traffic flow and/or safety dilemma in urban settings. To improve bicycle traffic flow through a busy Amsterdam intersection, the traffic signals were removed in 2017 at Alexanderplein in a bid to reduce unexpected manoeuvres from bicycle users and pedestrians that disobeyed the traffic signals. This is a useful example in demonstrating that low-speed ‘negotiation in motion’ (Jensen 2010) is not only safe but can improve traffic scenarios (Hahn and te Brömmelstroet 2021). It is also a case of how the *removal* of technology can improve outcomes. Negotiation is a weakness of AI-enabled mobility when the state-of-the-art solution is an HMI device. Recent legislative proposals in the United States seemingly signal the intent to advance HMI technology, which we caution as a potentially detrimental precedent for VRUs and organic urban movement/street life (Reid 2021b). The ‘beaconization’ of street interaction and movement offers great potential for eliminating street conflict, however, it presents a scenario where society would need to accommodate technology, rather than the technology accommodating society (Rupprecht et al. 2018). This presents existential questions for the vision of the AI-enabled city, equity questions due to the digital divide, and accessibility questions for those who rely on adaptive technology.

Conversely, other emerging technologies have the potential to strengthen the offering of AI-mediated mobility for VRUs. Although the operation of active and micro-mobility transportation means may not be as readily AI-enabled, other applications can make bicycles more attractive, accessible, and acceptable to a broader user base. For example, where fleets of connected smart bicycles or smart docks are deployed, AI can work to balance the availability of bicycles depending on a multitude of factors such as time of day, weather, special events, and elevation. There are even broader applicability scenarios where mobility as a service (MaaS) suites enable individuals to combine modes of transportation and bundle route planning into a single price/application. While these applications show promise, they may have little effect when the dominant infrastructure available in urban transportation systems induces demand for automobility by default (Sarker et al. 2002). Depending on how and where AI is applied to mobility in cities, the technology can ‘make or break’ the vitality of urban transportation. Any advancement of AI technology must be taken with humility and regard for holistic outcomes, especially for VRUs.

Fully developed, mass-market-ready AI-enabled mobility has the potential to eliminate human error which contributes to 93% of road crashes (Salmon et al. 2010). This is a valuable benefit of AI systems for creating a drastically safer environment for cycling and other VRUs. This advancement could lead to much lower perceived cycling risk and contribute to a safer environment, should AVs and AI-enabled mobility avoid another oppression. In addition, if AI-enabled mobility is to be *shared* (as in shared autonomous vehicles (SAV)), this may compromise the system of automobility’s intense flexibility (Urry 2004) and individuality. In this case, the bicycle may afford the most flexibility for urban mobility, especially for short trips.

In an age of autonomous mobility, there exists the potential elimination of all eye contact and face-to-face interaction. These are both essential in confirmatory communication for VRUs that give them confidence that their intentions were interpreted correctly. Although communicating to a driver through a windshield using eye contact and hand signals is prone to a degree of error, the trust and behaviour of cyclists and other VRUs stands to be completely eroded without adequate two-way communication from an AV. AI is a non-biological intelligence that functions differently from human beings (Cugurullo 2020). We cannot expect an AV driven by an AI, to operate like a conventional car. AI is a faceless technology that is not going to establish eye contact with cyclists, and there is thus a clear disconnect between the way cyclists communicate via face-to-face interaction and the physiognomy of AI.

Another contributing factor to bicycle oppression is the burden of responsibility in jurisdictions that do not have laws for presumed liability. In pitting multi-tonne projectiles (automobiles) against cyclists, there exists an unequal power dynamic (Carruthers 2017). The burden of responsibility should fall on the entity that inflicts harm (Mols 2017). Presumed liability laws are a legislative tool that can lessen the burden on vulnerable cyclists. In the event of a crash, the liability is automatically assigned to the motorist (Maker 2015). However, presumed liability is not universal. While most European countries have presumed liability laws, five countries (Malta, Cyprus, Romania, Ireland, and the UK) remain outliers and continue to assign the burden of proof to the VRU. There are larger questions around liability for inflicted harm when AVs become more prevalent (Awad et al. 2018; Gogoll and Müller 2017), however, presumed liability remains a tool that should be used more extensively and strengthened to protect and encourage cycling. There may be situations where AI will face ethical dilemmas and need to take an action in ‘trolley problem’ situations (Cugurullo 2021). As a result of these actions, it will need to be determined who will be liable. These novel dilemmas necessitate changes to legislative frameworks, particularly around presumed liability, so that cyclists and other VRUs are not left without options should they choose to seek damages for inflicted harm. When viewed holistically, existing literature at the nexus of AI, autonomous vehicles, cycling, and urban infrastructure makes clear that there are a multitude of considerations, challenges, and possible solutions. Furthermore, these developments are and will continue to be shaped by local jurisdictional authorities that implement them.

## Case studies on the rise and fall of bicycle oppression

In understanding the dynamics at play with an imminent future of AI-enabled mobility, there are two examples of societies that are synonymous with bicycle use, Denmark and the Netherlands. These societies were prompted in two different ways to adopt a ‘new normal’ following a period of austerity, and a social movement (respectively). In looking at these two examples, we can extract lessons and strategies for how to best pre-empt AVs in cities. While the Danish and Dutch were able to take a reactive approach to diversify their transportation systems by prioritising the bicycle, another approach would be proactive. We argue that in learning from these examples, an intentional reprioritisation of active transportation during times of stability can result in better outcomes.

### The Danish case

Danish bicycle culture arose out of necessity and in tandem with economic austerity measures during the energy crisis in the 1970s. In line with Danish folklore and the Law of Jante (see Sandemose 1933), the bicycle was conveniently compatible with egalitarian principles of social democracy during that time (Gulsrud 2019). The apparent lack of a domestic auto manufacturing industry (and lobby) also made top-down measures to reduce car dependency much easier to implement (ibid).

In the early 1900s, bicycling was common in Denmark. Danish lawmakers enacted a car tax during this time that reflected the view at the time that a car was a luxury item. Although unintentional, this tax served as an effective car restraint measure in the Danish capital, Copenhagen. After its introduction in 1910, the vehicle registration tax was increased in 1920 (Gulsrud 2019). Today the tax still applies in three brackets, where cars are taxed 25% on values of up to DKK 65,800 (approx. 10,000 USD or 8800 EUR), 85% on values between DKK 65,800 and 204,600 (approx. 10,000–31,000 USD or 8800–27,500 EUR) and 150% on the rest of the value (Danish Customs and Tax Administration 2022).

Despite this, by mid-century, the Danish capital was planning for “large parking structures and assumed that motorways would be forthcoming” (Gulsrud 2019, p. 57) and bicycle users were encouraged to “switch to public transit in order to make room on the streets for cars, and some cycle tracks and bicycle paths were removed” (ibid). At the height of bicycle oppression, bicycles made up 17% of all trips while cars made up 30% (Oldenziel et al. 2016).

As the energy crisis unfolded, Danish progressives were well-positioned to react as they articulated transport policies “away from automobility and towards green mobility” (Gulsrud 2019, p. 58). In a bid to reduce car and oil use, the national car tax and VAT were raised. The energy crisis led to Copenhagen’s deindustrialisation. The tax base used to fund major infrastructure projects left the city and bicycle infrastructure was the most cost-effective way to invest dwindling revenue. Given the economic situation, motorways and urban renewal projects were dismissed for more frugal bicycle projects. The reclamation of bicycle space can not only be attributed only economic circumstance in the Danish capital, but also through the deliberate action of an organised left and an extension of social democratic values embodied by bicycles (Gulsrud 2019).

Although Copenhagen has achieved a high cycling mode share, it was a reactionary response to a dire situation and a socio-political movement. Today, the Danish success story is used as somewhat of a greenwashing spectacle that was funded with a compromise of social democratic values enabled by neoliberal funding mechanisms to gentrify much of Copenhagen’s harbour (Gulsrud 2019). Without deliberate action prompted by the energy crisis and a socio-political movement, the top-down measures to bring back the bike would not have materialised. While the bicycle has made a comeback in Denmark, blindly advancing AV technology at the expense of all other modes would create a relapse of auto-exclusive planning that existed for a period and led to the oppression of other transportation, such as bicycles.

### The Dutch case

While the energy crisis had some impact on bicycling in the Netherlands, cycling has always been part of the national habitus (Kuipers 2013), but it was severely curtailed in the mid-twentieth century. Dutch car ownership grew from 14 cars per 1000 people in 1950 to 300 per 1000 people around 1980 (Reid 2017). During this time, bicycle mode share fell in the Dutch capital from 60 to 35% in 1973. There was virtually no mention of bicycles from the Dutch Government’s vision between 1950 and 1975. While the rate and use of car ownership grew and severely encroached on bicycle use, cycling was still accepted as normal. The extensive network of bike infrastructure meant that bicycle oppression was significantly less severe than in other countries (ibid).

A major turning point was the social movement called ‘stop de kindermoord’ or ‘stop the murder of children’. The movement was sparked by an op-ed with the same title written by a journalist who had one child killed and another injured by a motorist. By 1971, “Dutch motorists killed 3,000 people, 450 of whom were children” (Reid 2017, p. 197). The movement helped to advance even better Dutch bike infrastructure such as the woonerf, the car-free centre-city, and car bottlenecks. Stop de kindermoord offered practical solutions and paid for the salary of a traffic engineer with the Dutch Government to advance their cause.

The Netherlands began implementing the recommendations from the stop de kindermoord movement to build out high-quality bike infrastructure in the 1970s and has left a legacy where cyclists have dedicated space (Babb 2021; Reid 2017). This dedicated space resulted in clear delineation between bicycles and cars that is easy for AV sensing technology to interpret. This may position the Netherlands to avoid the next oppression cycle.

Like Denmark, there existed no Dutch automaker. Therefore, the social movements that reversed bicycle oppression and created countermeasures for car traffic were not met with opposition from domestic auto lobbies.

## How can bicycle oppression be avoided in AI-mediated transport systems?

As illustrated by the examples in Denmark and the Netherlands, there is no recipe to prevent future cyclical transport oppressions. In broad strokes, we can remark that the two cases previously discussed involved differing *temporal* and *sociopolitical* components. From a temporal standpoint, both cases deal with reactive measures to roll-back bicycle mode oppression; that is, both enacted measures to reverse the most recent transport mode oppression, something that is rare on the scale that was implemented in both cases. The sociopolitical contexts, however, differ in both situations. On one hand, Danish bicycle urbanism arose out of austerity measures during the energy crisis and took a more top-down approach that involved buy-in from Danish society, which was aided by the efforts of advocacy groups. On the other hand, Dutch bicycle urbanism came to exist through the work of advocacy organisation ‘stop de kindermoord’ which orchestrated a grassroots, bottom-up approach that needed buy-in from government. In each case, the reactive measures were responding to a crisis that could have been avoided through proactive measures. In understanding these cases, and the potential future crisis that AI-mediated transport systems may inflict on our cities, using the knowledge of the Danish and Dutch examples, cities can use foresight to avoid another cyclical mode oppression in a future with AI-mediated transport systems and a related crisis. However, in both cases, bicycle use was widely regarded as a transportation mode worth investing in and planning for.

To this end, sentiment toward cycling has a role to play in how it is integrated into the transportation system. As such, the prevailing sentiment and use of cycling is as a recreational activity (Heesch et al. 2012). Cycling needs to be considered a transportation mode with utility for it to gain credibility in the transportation system like how it is treated in the Dutch and Danish psyche. Shifting the collective psyche presents a significant challenge because of the deeply embedded preconceived notion of the system of automobility. However, we argue that the deployment of AI in urban transport systems is likely to reshape the collective psyche anyway. In this mutable cultural context, the role and perception of cycling might radically change. For example, one possible scenario is represented by the Volvo 360c: an AV that is depicted by Volvo as a space of pure leisure where the passenger can party, sleep, or simply enjoy the view while the AI is driving (Cugurullo 2021). If AI will redefine cars as recreational spaces, bicycles as a form of active transport might emerge in the future as one of the few means of urban transport in which humans are not simply passengers seeking leisure.

The reduction of risk and provision of safe cycling facilities in urban areas such as in Denmark and the Netherlands can help to promote cycling as a mode with utility. Programs that fund and include infrastructure for cycling have good potential for diversifying transportation mode share. In many cases, recreational cyclists are a key demographic that could easily transition to cycling for utility (Boyer 2018; Heesch et al. 2012) and the barrier that exists is the lack of safe and comfortable cycling facilities (Cabral and Kim 2020; Dill and McNeil 2013). Contemporary measures have made strides to shift the transport power dynamic away from automobility. In the 2010s, New York City’s transportation director, Jeanette Sadik-Kahn, was one of the early leaders that brought safer, human-scale urban cycling and public spaces to New York inspired by her experience in Copenhagen (Reid 2017) which marked a shift in how bicycles were designed for through fact-finding and data-driven decision making (see Sadik-Kahn and Solomonow 2017). London’s Mini Holland scheme, based on Dutch bike infrastructure design, is another example of proactive infrastructure planning and design can lead to favourable outcomes for bicycle users (Aldred et al. 2019). However, neither of these measures matches the scale or responds to the exigency that Danish and Dutch bicycle planning faced in the 1970s.

More recently, programs that have been enacted to help shift public sentiment toward cycling as a utilitarian transport mode include initiatives that increase the visibility and lower the risk of cycling. Both the Dutch and Danish used car-free days which are now commonplace in cities like CicLAVia in Los Angeles, Open Streets in Toronto, Summer Streets in New York City, Car Free Day in Vancouver, or through pop-up initiatives like Stockholm’s summer streets program. During the COVID-19 pandemic, cities like Dublin’s Capel Street pedestrianization, Calgary’s Memorial Drive space reallocation, and Boston’s Healthy Streets program have given pause to how residents think about how their city’s streets can be used.

Shifting transportation infrastructure would be a costly and time-consuming undertaking and, while automobility remains dominant, there will be a constant strain on finite space in urban areas, particularly as it pertains to the staging (pick-up/drop-off) of passengers at the curb. One of the most effective tools for curbing car-related transport capacity problems is to use roadway pricing (Ubbels and Verhoef 2007). An early example of using AI to mediate this interface and staging for curb use can be found in the SFPark system that dynamically prices on-street parking to maintain a fixed level of availability using embedded sensors (Chatman and Manville 2014). Solutions like this could be part of a strategy to maintain the integrity of curb space and redirect demand to less congested areas, thereby encouraging the use of alternative transportation. This redirection may encourage the use of public transit or urban cycling to complete the last mile of a journey in areas where there is increased demand for curb space.

Transportation planning practice can be divided over the end-state for AI-enabled mobility. On one hand, the future of transportation can become devoid of urban life and be used exclusively for vehicular movement. On the other hand, AI-enabled mobility can seamlessly interpret organic urban life without the need for an HMI. The approach that is advanced will have significant implications for the vitality of cities and urban life as it exists today. Cyclical transportation oppressions, as discussed in this paper, can lead to a pseudo-sanitization of streets—devoid of human activity in the interest of vehicular movement. If autonomous mobility is pursued aggressively, this oppression could be the most drastic to date. Urban areas are centres of human-scale activity and require a different frame of mind when considering all the activity that exists on a street. Advancing a vehicular movement-first approach could cause irreversible damage to the street life that makes cities vibrant social hubs. Given this, we argue that AI-enabled mobility must reach a more mature state before it is aggressively deployed.

The variety of actors on urban streets and public spaces are responsive. People on bikes are among street users that are incredibly adaptable and communicative. Jensen described the ability of ‘negotiation in motion’ in their 2010 paper as the ‘river’ and the ‘ballet’, drawing on work by Jacobs (1961). Other leading urban thinkers have corroborated that people respond to their environments and urban life thrives when they are designed using human-centred design principles (see Whyte 1980; Gehl 2011). The effect on livable urban spaces cannot be achieved with the singular mission of vehicular movement is the goal. This is a key lesson that must be taken into consideration when planning for AI-mediated transport systems: they must *evolve* urban life to a more livable state; not *devolve* it into a harsh and unwelcoming environment. The Dutch and Danish cases are examples of how auto-oriented planning and design prompted decisive action that made a deliberate change in favour of a more human urban environment.

## What can be done to avoid further oppression in an age of autonomous transport?

The spheres of influence over transportation cycles span across the political, social, and design dimensions. In each sphere, there are roles for policymakers, researchers, and both working in collaboration. The synergy of research and evidence informing policy is exigent in developing an equitable, efficient, and sustainable transportation system with AI-enabled mobility. In addition to the roles that the identified actors in Table 1 below can play in each sphere, there are key actions at the intersection of transportation and land use that must occur for a future cycle of mode subordination to be thwarted. The three spheres of influence are discussed in more detail below.

**Table 1:**
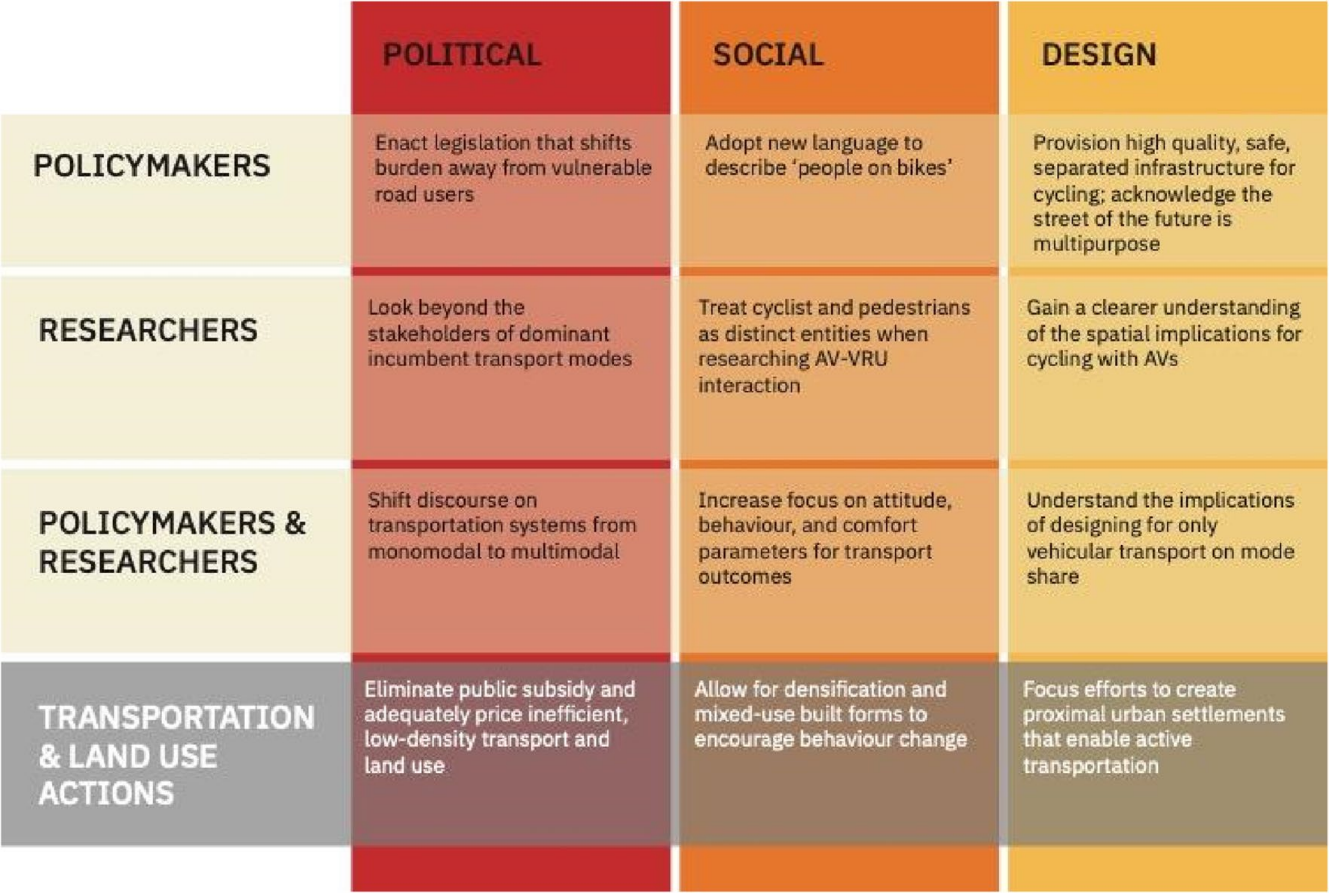
Summary of actions for policymakers and researchers to break the transport oppression cycle in the age of AVs

### Political

Policymakers and researchers have a significant role to play in how the next era of transport unfolds. Both can work together in a similar way to how the Danish and Dutch transitions were informed by external forces. The contemporary city and autonomous mobility will continue to evolve based on the discourse laid out by policy and research. Emphasizing technology first and incumbent dominant modes of transportation will result in similar outcomes to historical transport oppression. Taking into consideration the underpinning factors and strategies to avoid another oppression will determine how cities and transport networks perform in the coming decades when AI is likely to mediate several urban transport systems. This is a high-stakes moment that can have lasting impacts as autonomous mobility moves from the pre-implementation phase to a more mature technology that increases in ubiquity.

The prevailing discourse and system of automobility will need to be shifted. A supportive and more balanced legislative framework must emerge to accommodate a wider variety of transport modes more fairly for those that have been historically oppressed. This includes reallocating street space for multimodal transportation that has been designated exclusively for vehicular transportation in the Modernist City (Creutzig et al. 2020). Accommodating all types of cyclist comfort by building street configurations with separation based on the lowest common denominator has good potential to yield results for all types of cyclists (Cabral and Kim 2020; Dill and McNeil 2013).

### Social

**Fig. 2:**
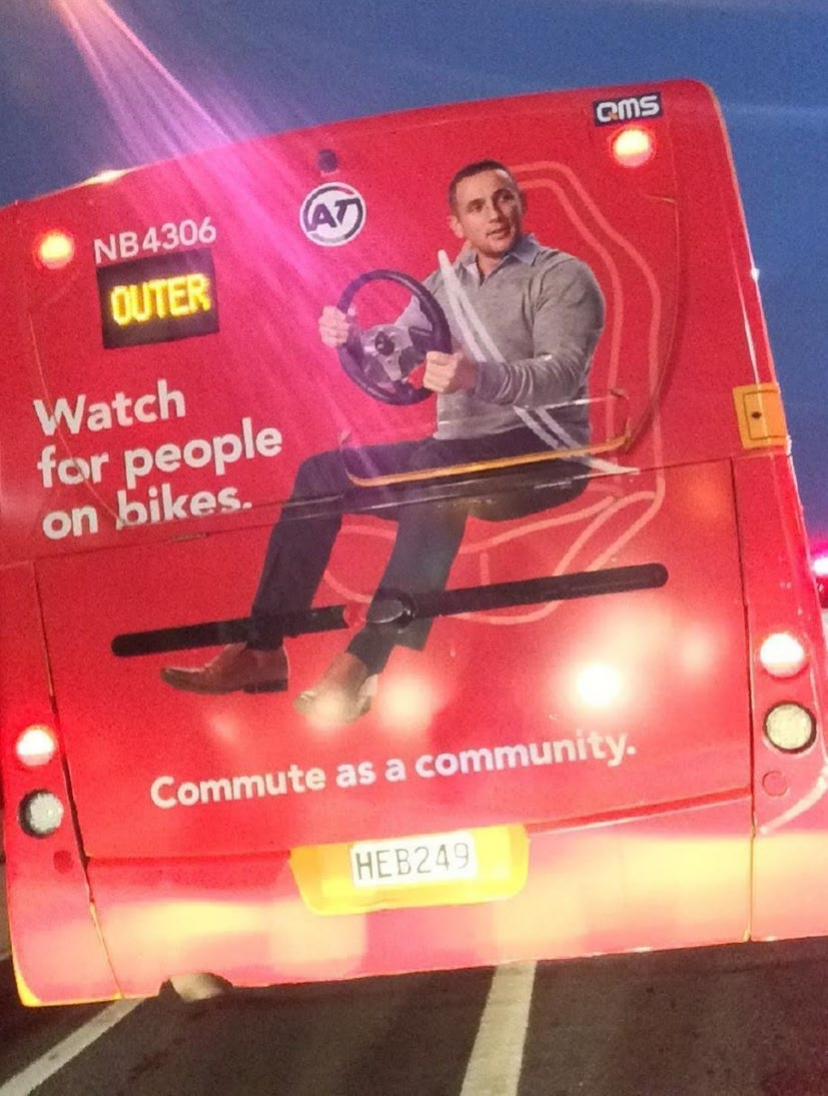
Auckland transport using ‘people on bikes’ terminology. Source: Authors’ original

Language and communication around descriptors will need to adapt. Although ‘cyclist’ and ‘motorist’ are used throughout this paper to match keywords that exist in the field, it has been suggested that this type of language creates division and does damage to people who ride bikes. A study by Delbosc et al. found that “Around half of non-cyclists [in their study] view cyclists as ‘less than fully human’" (2019, p. 681). They go on to explain that “if dehumanization of cyclists is occurring, this could be contributing to the hostility and aggression experienced by cyclists on the road” (ibid, p. 681). Some jurisdictions have changed how they advertise and describe how they refer to the act of riding a bicycle as ‘people on bikes’ (Fig. 2) in an effort to de-marginalise and create a more approachable, human image of ‘cyclists’. Moreover, as we mentioned previously, the diffusion of AI in urban transport systems is changing the very nature and perception of driving. For example, in a hypothetical future urban context dominated by fully AVs *à la* Volvo 360c, the term *motorist* would not be appropriate since the human driver would turn into a passenger under what Mokhtarian (2018) terms ‘passengerization.’

Alternative modes of transportation will need to be accommodated as pragmatic and utilitarian modes of transportation. This will further require the provision of high-quality separated cycling infrastructure. It is important, in an age of autonomous transport, that all groups of cyclists must be considered including groups such as MAMILs (middle-aged men in lycra), commuter cyclists, and utility cyclists (Aldred and Dales 2017). The latter category is a key demographic to plan for because they can be an indicator demographic that exists on the less confident end of the cyclist spectrum (Dill and McNeil 2013). Furthermore, street layouts should be reconfigured to allow for transportation choice, ‘vision zero’ traffic fatality elimination commitments,^1^ and overall safety maximising the movement of people instead of vehicles. Metrics of success will need to shift away from vehicle-centric metrics toward maximising movement of people.

### Design

There will also be a significant role for the intersection of transportation and land-use policy to counter an ever-expanding urban footprint. As AI-mediated transport in the form of AVs stands to remove the burden of driving, there is a real possibility that longer commutes could become desirable (Cugurullo et al. 2020). All else being equal, if longer commutes become acceptable, the cost to service the expansive transport infrastructure and municipal servicing will become cost-prohibitive (Slack 2002). The costs for such expansion need to be borne by individuals who opt for such commute distances. This ‘growth pays for growth’ model through development charges should fully pass the cost of exurban development onto the individual instead of subsidizing it using public funds (ibid). In creating a longer commute, this enables a more dispersed and low-density settlement that becomes even more difficult to serve by higher order rapid transit and continues to marginalise cycling and other modes of active transport as practical options (Burda et al. 2012). In a period of climate change, there is a need to create more resource-efficient and proximal settlements that do not encroach onto natural areas (ibid). AV super-commuting would serve as the antithesis of the (cyclable) 15-min city which has been touted as a solution to excessive automobility in cities.

Land-use and transportation policies will face existential challenges to contain growth within existing urban boundaries and to develop nodal cities that have amenities for enabling transportation choice instead of predicating prosperity on the assumption that a vehicle is a prerequisite to have a high quality of life. The prevailing global pandemic has also shone a light on society’s ability to stay in place and has highlighted the need for proximal amenities, further strengthening the case for a 15-min city (Moreno et al. 2021). Low-density environments that rely on the system of automobility tend to be obesogenic (Lake and Townshend 2006). The nature of living a lifestyle that depends on extended periods of stagnation (e.g. commuting) is not conducive to individual or public health outcomes. Over the last several decades, physical activity has been engineered out of everyday life, and as people re-join the daily ebbs and flows of daily transport patterns, there will be a need to reconsider how physical activity can be planned back in. Cycling alongside AI-mediated transport systems in a proximal urban environment could be a solution to this dilemma.

While early research on cyclists and motorcyclists suggests that cyclists trust AVs more than human drivers, only 5% of the respondents involved in a study by Pammer et al. (2021) were identified as only cyclists and it is difficult to generalise findings from a small sample size. Cavoli et al. (2017) have also written that trust AVs more than human drivers when they are programmed to operate safely, but only law-abiding VRUs have more trust than those that do not obey traffic laws. In a paper by Hagenzieker et al. (2020, p. 110), it was found that cyclists may have “incorrect or over-reliant expectations of automated driven cars” which may impact mutual trust if AVs are programmed to operate safely, but subsequently programmed to operate more aggressively to assert their place in the road. This may lead to erratic cyclist behaviour and is difficult to predict using hypothetical scenarios without empirical real-world research outside of simulations.

Further to Hagenzieker et al. the gap in research intersecting the topics of AVs and cyclists was a demonstrable omission that needed to be resolved to better understand the implications of AVs on cycling (Ahmed et al. 2019a, b; Botello et al. 2019; Coelho and Guarnaccia 2020; Eldesokey et al. 2017; Kress et al. 2018; Penmetsa et al. 2019; Wang and Akar 2019; Zhang et al. 2020). Of note is the fact that, while there is research on AVs, the primary research focus is either on hypothetical future users or is too thematically broad to draw adequate conclusions for cyclists. These broad conclusions are frequently grouped into a universal codification of ‘vulnerable road users’ or VRU. VRUs are defined as a “non-motorised road users such as pedestrians and cyclists as well as motorcyclists and persons with disabilities or reduced mobility and orientation” (European Union 2009, no page). This codification is not specific enough to adequately distinguish cyclist characteristics from those of other VRUs.

Other recent studies have found that, related to trust, communication between AVs and cyclists is still underdeveloped. Communication gaps are likely a major contributor to a lack of trust between road users. In a paper by Botello et al. “[n]early half of the interview respondents raised the issue of communication between C/AVs and cyclists and pedestrians” (2019, p. 4). Some respondents suggested that, while more explicit communication from AVs may be helpful, their ability to detect subtle signals, hints, or even no signals was a weakness in the technology’s functionality in real-world traffic scenarios involving cyclists and pedestrians (ibid). Subtle signals such as waves and direct eye contact are important for establishing trust and understanding between road users in high-pressure or high-stakes interactions. When a cyclist's hands and feet are engaged, the only effective form of communication they can use is eye contact. This was also corroborated in a study by Hagenzeiker et al. where “[n]onverbal communication between road users will become more or less useless” (2020, p. 97) when communicating with AVs.

Much of the existing research around AVs concerns the occupant’s perspective (Rahimi et al. 2020; Saeed et al. 2020). Other research broadly addresses the opinions and preferences (Cugurullo et al. 2020), or the moral challenges around AI morals for decision making (Awad et al. 2018). While there are several other themes, they all concern addressing the novelty, instead of considering the impact of the technology on the perspective of road users that have been systematically oppressed through previous novel transport technology. Furthermore, little emphasis is placed on the cyclist’s attitude, behaviour, or infrastructure needs as cycling continues to make a larger share of transport (Reid 2017) both before and during the COVID-19 pandemic (Buehler and Pucher 2021a) and with more net positives than automobility in cities (Brand et al. 2021). This calls for prompt action to better understand the implications AVs will have on this specific subset of VRUs—bicycle users.

In 2006, the bicycle coordinator at the City of Portland proposed a way of classifying cyclists according to their level of comfort. This classification system has been influential in cycling research and policymaking (Dill and McNeil 2013). Others have used classification systems that are based on frequency of cycling (Heinen et al. 2011; Winters et al. 2011), seasonality (Bergström and Magnusson 2003), and cycling stereotype (Gatersleben and Haddad 2010). While these classification systems that academics have developed are useful for classifying cyclists, they are somewhat inward-looking and do not encompass a perspective of the population that may fit into categories if they consider themselves a ‘cyclist’ or not (Dill and McNeil 2013).

Under conventional traffic scenarios “[t]he increased separation [of bicycles] from traffic is thought to improve the level of comfort” (Monsere et al. 2012, p. 22). Further to questions of comfort and attitude, the street design and safe infrastructure needs of cyclists in an autonomous mobility future will dictate how our cities and surface transport systems are designed. This is pertinent because AVs may eliminate the need for parking and wide road lanes, freeing up space for other uses (Duarte and Ratti 2018; Santana et al. 2021). There will be competition for any potential available space that may arise due to the nature of its scarcity.

However, research on bicycle facility preferences whilst riding with AVs is scarce. While some research suggests that there is a need for more separated cycling infrastructure, some scholars have suggested that VRUs may consider AVs more trustworthy than human drivers (Pammer et al. 2021) which suggests less of a need for AV–cyclist separation. Conversely, this trust may not be sufficient to double-back on safe separated cycling infrastructure as there will likely continue to be the need for it in the future (Botello et al. 2019), especially if ‘comfort’ is a defining factor in the predictors of cyclist behaviour.

## Conclusion

The cyclical oppression of legacy transportation modes has been a recurring effect of transportation technology advancements. The era of the Modernist city ushered in a new paradigm for mobility that focussed primarily on automobiles and excluded all other transportation modes. The oppression of non-auto modes has similar links to previous transport mode subordination throughout history. A repetition of the historical transport mode oppression could be an outcome in an AI-enabled mobility future if steps are not taken to ensure the auto-oriented transport paradigm is not shifted towards a more inclusive and multimodal transport future. This shift will require engaging researchers and policymakers to address political, social, and infrastructure design realms to create a more efficient, equitable, sustainable, and healthy transportation system for the next era of transport innovation.

Recently, a worrying sign of a looming *third oppression* could be seen at COP26 2021 in Glasgow. The most effective and efficient way to decarbonise urban transport, bicycle transport, was absent from the discussion and was added as a last-minute addition to the conference’s declaration (Reid 2021a, c). Addressing cyclical transport oppression in the age of autonomous transport will take deliberate action. It will involve a multifaceted systems-based approach at different scales to confront transport mode oppression. There are three primary areas in which this oppression occurs: the political, social, and infrastructure design (Table 1). A combination of addressing the core factors of cyclist oppression and working to build a transport system that works for all transport modes instead of only (autonomous) vehicles will result in better outcomes for transport network efficiency, population-level health, and GHG emissions.

The perspectives and critical appraisal of AI-mediated mobility systems outlined in this paper are meant to galvanize the debate around AI-enabled mobility and bring new topics to the forefront. In doing so, we hope that this adds nuance to the rapidly maturing technology that will enable AI-mediated mobility to become reality. Furthermore, we also hope that the urbanism literature, transportation planning literature, and background information shared in this paper are new and help to bring new interdisciplinary perspectives.

While cycling is not the only transport mode that stands to be impacted by AI-mediated transport systems, transportation and land use policies will need to work to prevent the next oppression not only of cycling, but of public transportation, and other modes. AI-enabled dynamic roadway/parking pricing, and development cost recovery will help to attribute the full cost of urban growth to new developments. Transportation, land use, and (dis)incentives will need to be used to embody the full cost of AI-mediated transport. It is also important to avoid complete privatisation of the transportation system.

This paper has argued that, based on historical events and prevailing trends, that there may be a looming threat to mobility choice and a *third oppression* in a future of AI-enabled mobility in the form of the AV. There are broad societal implications that a *third oppression* and stifling of transportation options would have on outcomes related to climate change, public health, and traffic congestion (Garrard et al. 2012; Götschi et al. 2016; Kelly et al. 2014; Macmillan et al. 2014; Oja et al. 2011). However, there are further emerging concerns around how AI is adopted and how it influences transportation systems and, in turn, mobility justice (Sheller 2018). In recalling how the most recent transport oppression pitted automobiles against all other modes, there existed a strong automobile lobby to advocate for a political context in its favour. The current “politics [of AI] are driven by Great Houses of AI, which consist of the half-dozen or so companies that dominate large-scale planetary computation” (Crawford 2021, p. 20). This concentration of power, not dissimilar to that of the previous oppression, could lead to a similar political context in favour of AI-enabled mobility and lead to a *third oppression* as we have outlined and warned in this paper.

## Data Availability

Not applicable; no new data were created for this publication.

## Footnotes

1 ‘Vision Zero’ is an international strategy to reach the goal of eliminating all traffic fatalities and a novel approach to traffic safety.
